# Effects of Age and Season on Blood Parameters of Captive Giant Pandas: A Pilot Study

**DOI:** 10.3390/ani13193023

**Published:** 2023-09-26

**Authors:** Ruijie Jiang, Xinyi Zhang, Maohua Xia, Sufen Zhao, Yunsheng Wang, Tianchun Pu, Chenglin Zhang, Zhong Wu, Haihong Xu, Kai Fan

**Affiliations:** 1 College of Veterinary Medicine, China Agricultural University, 2 Yuanmingyuan W Rd, Haidian District, Beijing 100091, China; jiang6494@alu.cau.edu.cn (R.J.); sy20213050936@cau.edu.cn (X.Z.); 2 Beijing Zoo, 137 Xizhimenwai Street, Xicheng District, Beijing 100044, China; xiamh@sohu.com (M.X.); zhaosufen111@126.com (S.Z.); wangyunshengabc@163.com (Y.W.); putianchun0628@sina.com (T.P.); zhang_chenglin01@sina.com (C.Z.); jrj20632312@163.com (Z.W.); 13911619616@163.com (H.X.)

**Keywords:** giant pandas, seasonal, age, blood indices, hemorheology

## Abstract

**Simple Summary:**

The giant panda (*Ailuropoda melanoleuca*) is a flagship species for biodiversity conservation. Due to population size and sample collection limitations, very limited information is available on the routine blood and blood biochemistry of giant pandas. Rarely is the influence of seasons on the physiology and health of giant pandas studied. Understanding the impact of meteorological factors on giant pandas is necessary for the future. In this study, we obtained data on the changes in hematological parameters of giant pandas in different seasons and ages. We found differences in overall metabolic processes among giant pandas of different age groups. At the same time, seasonal changes have a regulating effect on hemorheological parameters and may have a negative impact on blood viscosity. Whether seasonal and climatic factors cause environmental stress in captive giant pandas remains to be further studied. The results of this study may help to protect the stability of the giant panda population better and provide a reference for the appropriate feeding and medical care of captive giant pandas.

**Abstract:**

The giant panda, *Ailuropoda melanoleuca*, serves as a flagship species for biodiversity conservation, embodying the intersection of ecological, evolutionary, and anthropogenic forces shaping the natural world. Hematological parameters serve as crucial indicators for assessing the physiological status of animals. However, our understanding of blood parameters and hemorheology in captive giant pandas under non-anesthetic conditions is limited. In this study, from September 2018 to August 2020, we collected blood samples from captive giant pandas under non-anesthetic conditions. Twelve captive giant pandas, ranging in age from 2 to 28 years, were divided into three groups based on their age, and the variations in basic blood parameters and hemorheological parameters across four seasons were analyzed. This provided baseline data for future blood sample comparisons in non-anesthetized captive giant pandas. Additionally, we observed seasonal changes in hematological morphology, hemorheology, and serum enzymes. Moreover, seasonality had a regulatory effect on hemorheological parameters and negatively impacted blood viscosity. Age influenced changes in serum enzymes, serum protein content, and serum metabolites, indicating differences in overall metabolic processes among giant pandas of different age groups. Whether factors such as season and climate contribute to environmental stress in captive giant pandas requires further investigation. The findings of this study may help to protect the stability of the giant panda population better and provide a reference for the medical care of captive giant pandas.

## 1. Introduction

The giant panda (*Ailuropoda melanoleuca*) serves as a flagship species for biodiversity conservation, embodying the intersection of ecological, evolutionary, and anthropogenic forces shaping the natural world. It holds significant academic research value on a global scale [1,2,3]. However, due to population size and sample collection limitations, there has been a limited number of reports on the blood routine and blood biochemistry of giant pandas. Blood variables are crucial for understanding the impact of diseases on individuals and populations, as well as assessing the health status of individual animals [4]. Some reports have shown that certain hematological variables in giant pandas are influenced by gender, age, physical condition, season, and region [5,6,7,8]. However, due to the unique characteristics of the giant panda population, there are some limitations in exploring aspects of the blood analytes of giant pandas. This study is a report on blood variable measurements in captive giant pandas obtained without anesthesia. It includes parameters such as morphology, hemorheology, serum enzymes, serum proteins, and serum metabolites of captive giant panda blood. The purpose of this study is to describe the age and seasonal changes in blood parameter variables in captive giant pandas in Beijing, China. The aim is to protect the stability of this population better and provide references for the rational feeding and medical care of captive giant pandas.

## 2. Material and Methods

### 2.1. Animal Location

In this study, an equal number of male and female giant pandas aged between 2 and 28 years were included. They showed no abnormalities in their eating, drinking, or excretion two weeks before blood collection. The pandas were housed at the Beijing Zoo, with geographical coordinates at 39.9° N, 116.3° E. The basic information about giant pandas can be found in Table 1 and Table 2. The bamboo species primarily provided to giant pandas at the Beijing Zoo include *Phyllostachys propinqua* (mao bamboo) and *Pleioblastus amarus* (qing bamboo), supplemented with bamboo shoots and mantou (steamed buns). There were no significant variations in the energy, carbohydrate, and protein intake of giant pandas across the four seasons.

### 2.2. Season and Climate

The study was conducted continuously for two years, starting from mid-2018 and ending at the end of June 2020. Based on the regular climate patterns in Beijing, each year was divided into seasons as follows: spring includes February, March, and April; summer includes May, June, July, and August; autumn encompasses September and October; and winter spans from November to January. Beijing, China experiences a temperate monsoon climate characterized by hot and rainy summers and cold, dry winters. During the experimental period, the average temperature in spring ranged from 1 to 18 °C with an average relative humidity of 29%. Summer had an average temperature between 19 °C and 30 °C with an average relative humidity of 45%. Autumn had an average temperature range of 17 to 29 °C with an average relative humidity of 52%, and winter had an average temperature range of −3 to 6 °C with an average relative humidity of 37%.

### 2.3. Blood Sampling

Twelve giant pandas underwent monthly blood collection procedures under operant conditioning. All participating pandas had successfully completed positive reinforcement training. On the day of sampling, the pandas were fasted from midnight until 8 a.m. The blood samples were collected from the median vein in the front limb of the giant pandas, with each sample totaling 5 mL. These samples were placed in EDTA (Yuli, Jiangsu Yuli Medical Equipment Co., Ltd., Taizhou City, China) anticoagulant tubes for storage and were subjected to blood analytes within a two-hour timeframe.

### 2.4. Sample Treatment

The blood cell counts were determined using manual microscopy by trained laboratory personnel on a blood cell counting board. Hemoglobin levels were measured using the cyanide-free high-iron hemoglobin method. Red blood cell hematocrit values were determined using the Winslow method. Blood biochemical parameters were measured using the FUJI DRI-CHEM NX500iVC, a fully automatic dry chemistry analyzer made by FUJIFILM, Tokyo, Japan.

Hemorheology parameters were assessed using the HT-100A blood rheological viscosity tester produced by Zibo Hengtuo Analysis Instruments Co., Ltd., Zibo City, China. Whole blood viscosity was measured at shear rates of 3 S-, 30 S-, and 200 S-. Plasma viscosity was determined after centrifugal separation of anticoagulated blood.

### 2.5. Environmental Temperature and Body Temperature

Data from September 2018 to August 2020 were compared. Independent sample *t*-tests were conducted based on the monthly average temperature provided by the observatory. Due to multiple comparisons, the Bonferroni adjustment was applied to the significance level. Each giant panda’s body temperature was measured using an infrared ear thermometer inserted directly into the ear.

### 2.6. Statistical Analysis

The collected data were analyzed using GraphPad Prism software (https://www.graphpad.com/features) for statistical analysis. The article employed repeated measures analysis of variance (ANOVA) for statistical comparisons and conducted multiple comparisons using Fisher’s protected least significant difference (LSD) test. A *p*-value less than 0.05 was considered statistically significant.

According to the Shapiro–Wilk test, the data from each group were found to follow a normal distribution. However, the sphericity assumption test results indicated that the assumption of sphericity was not met. Therefore, the corrected results from the Greenhouse–Geisser test were used in the one-way analysis of variance.

### 2.7. Ethical Standards

This study was conducted on giant pandas, which are national treasures, and informed consent was obtained from the Beijing Zoo to collect data. During the research period, all animals underwent positive reinforcement training in accordance with official guidelines and regulations. These guidelines represent technical standards for the production, care, and use of (wildlife) animals.

## 3. Results

### 3.1. The Captive Housing Environment

Table 3 shows the outdoor living environment temperature and body temperature of giant pandas. The outdoor environment temperature in summer is significantly higher than in winter, with no significant difference between spring and autumn. The body temperature of giant pandas fluctuates in a regular pattern with the temperature of outdoor activities throughout the four seasons, increasing in summer and decreasing in winter. The indoor temperature is kept constant. In the experiment, giant pandas in a non-healthy state were not included.

### 3.2. The Hematological Parameters of Captive Giant Pandas

Season and age both affect the morphology of blood parameters in giant pandas. Table 4 shows that season significantly influences the levels of hemoglobin (*p* < 0.05) and RBC (*p* < 0.05). Compared to other seasons, blood parameters from giant pandas kept in winter exhibit higher levels of hemoglobin, RBC, and hematocrit. On the other hand, age significantly affects the levels of hemoglobin, WBC, RBC (*p* < 0.05), and hematocrit (*p* < 0.001). When compared to the blood parameters of older and younger giant pandas, the characteristics of adult giant pandas’ blood parameters show higher values for these parameters.

### 3.3. The Hemorheological Parameters in Captive Giant Pandas

The age of giant pandas does not affect (*p* > 0.05) the hemorheological parameters. However, the season does affect the hemorheological parameters of giant pandas (Table 5).

Seasonal conditions significantly affect the plasma viscosity (*p* < 0.05), erythrocyte aggregation index (*p* < 0.05), and erythrocyte rigidity index (*p* < 0.05). Compared to spring (1.69 ± 0.04 mPa·S; *p* < 0.05), higher levels of plasma viscosity were observed in blood parameters during summer (1.82 ± 0.03 mPa·S; *p* < 0.05) and autumn (1.86 ± 0.04; *p* < 0.05) in giant pandas. Additionally, the plasma viscosity measured in blood samples collected during autumn was significantly higher compared to winter (1.86 ± 0.04 mPa·S, 1.74 ± 0.03 mPa·S; *p* < 0.05). Compared to summer (6.73 ± 0.23), the erythrocyte aggregation index significantly increased in spring (7.63 ± 0.23; *p* < 0.05) and winter (7.82 ± 0.21; *p* < 0.05). The erythrocyte rigidity index was significantly elevated in spring (4.96 ± 0.18; *p* < 0.05) and winter (4.98 ± 0.12; *p* < 0.05) in giant pandas compared to summer (4.24 ± 0.14).

### 3.4. The Serum Enzyme Levels in Captive Giant Pandas

Both season and age have an effect on the serum enzyme parameters of giant pandas. The statistical analysis in Table 6 shows that seasonal changes significantly affect the serum enzymes of giant pandas: ALT (*p* = 0.001), CHE (*p* < 0.001), ALP (*p* = 0.01), and LPS (*p* = 0.01). Compared to summer, autumn, and winter, the spring season has higher levels of ALT, cholinesterase, and LPS (*p* < 0.01). In contrast, the ALP levels in autumn are significantly lower than in other seasons (*p* < 0.05). Age also has an impact on the serum enzymes of giant pandas: ALT, AST, cholinesterase, ALP, ADA, LD, α-HBDH (*p* < 0.001), and CK-MB (*p* < 0.05). The statistical analysis of the results also suggests a significant interactive effect of age on the seasonal variation in serum cholinesterase levels in giant pandas (*p* < 0.05).

### 3.5. The Serum Protein in Captive Giant Pandas

Table 7 shows that the age of giant pandas significantly affects (*p* < 0.05) the serum protein levels in their blood analytes, with sub-adult pandas showing lower levels of total protein and globulin. The season does not have an effect on the serum protein levels of giant pandas (*p* > 0.05).

### 3.6. Serum Metabolites in Captive Giant Pandas

Age did not have a significant effect (*p* > 0.05) on the levels of serum metabolites collected from giant pandas, while the season did not have an effect (Table 8). The bile acid level in adult giant pandas (3.88 ± 0.18 mmol/L) was significantly lower than in sub-adult pandas (4.59 ± 0.20 mmol/L; *p* < 0.05) and old pandas (4.20 ± 0.20 mmol/L; *p* < 0.05). The bile acid level in sub-adult pandas (51.35 ± 4.78 μmol/L) was significantly higher than in adult pandas (35.50 ± 3.89 μmol/L; *p* < 0.05). As for the serum uric acid level, old pandas (41.86 ± 2.50 μmol/L) had significantly lower levels than adult pandas (59.56 ± 3.48 μmol/L; *p* < 0.001) and sub-adult pandas (57.86 ± 1.62; *p* < 0.001).

## 4. Discussion

This study, by examining the age and seasonal variations in blood parameters of captive giant pandas in northern China, provides a baseline for future blood analyte comparisons in non-anesthetized captive giant pandas and offers valuable insights for the medical care of giant pandas in zoos. Based on the parameters collected in this study, if factors such as age and season are not considered, there are significant differences compared to the reference range of the pandas in Beijing Zoo. The main differences are reflected in the following: an increase in hemoglobin, red blood cell count, white blood cell count, and hematocrit; a decrease in blood glucose, total protein, albumin, globulin, triglycerides, uric acid, creatinine, total bile acid, alanine aminotransferase, aspartate aminotransferase, creatine kinase, cholinesterase, gamma-glutamyl transpeptidase, and amylase; and an increase in urea nitrogen, total cholesterol, lactate dehydrogenase, alkaline phosphatase, adenosine dehydrogenase, and alpha-hydroxy acid dehydrogenase. Analyzing the reasons for these changes, although the existing reference range in Beijing Zoo includes a total of 29 pandas, it also includes samples collected after anesthesia, and the influence of stress on blood analytes cannot be ruled out. Therefore, the reference range cannot be regarded as the average level of the tested animals. The blood samples measured in this study were all collected from non-anesthetized captive giant pandas. We hope that these data can contribute to enriching and enhancing the reference range for giant pandas at Beijing Zoo, providing valuable references for the blood parameters of pandas in the zoo in the future.

Specific lifestyles can lead to welfare issues in various wild animals kept in captivity, including poor health, repetitive stereotypical behavior, and breeding difficulties [9]. Analyzing hematological and biochemical parameters is an important part of assessing the physiological, nutritional, and pathological conditions of animals. Determining the levels of biochemical blood parameters is important for evaluating the physiological status of animal organisms as it enables the diagnosis of various diseases affecting internal organs. The reports by Deng and Luo et al. indicate that age significantly affects various hematological and biochemical indicators [10,11]. In this study, age influenced hematological parameters, hemorheology, serum enzymes, serum proteins, and serum metabolites. It was found that the elderly group of giant pandas had the lowest levels of glucose, hematocrit, hemoglobin, WBC, and urea as well as the highest levels of globulin, AST, CRE, and BUN, which may be related to the aging state of the giant pandas in Beijing Zoo or perhaps chronic stress. However, based on the analysis of ALT, CK, creatinine, and urea, all animal groups in the experiment showed normal values for these parameters; therefore, although the values of the elderly group of pandas showed significant changes, the animals were not in a diseased state. The alkaline phosphatase level in the sub-adult group was significantly higher than in the adult and elderly groups, which may be due to the fact that alkaline phosphatase can reflect bone formation and osteoblast metabolic activity, thus leading to higher levels in young animals. In clinical practice, we currently only know that the differences need to be treated accordingly, which can help clinical practitioners make correct and objective assessments when managing health [12].

This study found seasonal variations in hematological parameters, hemorheology, and serum enzymes. The seasonal changes in serum enzymes may be related to the spring estrus of giant pandas. Meanwhile, hematological parameters, such as HGB and RBC, were higher in winter. The plasma viscosity (*p* < 0.05), erythrocyte aggregation index (*p* < 0.05), and erythrocyte rigidity index (*p* < 0.05) showed significant seasonal variations. These findings may indicate a regulatory role of seasonal changes in blood viscosity in giant pandas. Clinical studies have found that many diseases are accompanied by abnormalities in various hemorheological indicators. Some of these abnormal indicators often appear earlier than symptoms or signs, and they often lag behind when symptoms or signs disappear during recovery [13,14,15]. Blood viscosity is mainly determined by red blood cell hematocrit, plasma viscosity, red blood cell aggregation, and red blood cell deformability [14,16]. Similarly, it has been shown that the effective viscosity of circulating blood increases as plasma osmotic pressure increases with age [17]. There is a significant difference in the red blood cell aggregation index between summer and winter. Therefore, the main reason for increased whole blood viscosity may be due to red blood cell aggregation. Studies have shown that ambient temperature (climate) has an effect on both blood viscosity and plasma viscosity in animals [18,19,20,21]. When the temperature decreases, blood flow slows down, blood viscosity increases, and red blood cell aggregation is enhanced [22,23,24]. This is because, during cold weather, animals need to consume a large amount of oxygen to generate heat to maintain a relatively stable body temperature. Inhaling cold air can also increase pulmonary artery pressure, leading to impaired gas exchange and tissue hypoxia, resulting in increased quantities of red blood cells [25,26,27]. This may explain the observed seasonal variations in blood parameters in pandas in this study. Additionally, the activity rhythm of many animals is significantly influenced by seasonal changes, and activity levels are directly related to temperature. When activity levels increase, the surface area of red blood cells relatively increases, which facilitates the release of oxygen from red blood cells and the removal of carbon dioxide from tissues, accelerating the gas exchange rate. This also reduces blood viscosity and increases blood flow velocity. However, there is currently a lack of seasonal research on the behavioral patterns or activity levels of giant pandas, and further related research is needed.

A successful ex situ conservation project has required us not only to save an animal species but also research the appropriate thermal environment for individual animal survival in order to develop corresponding conservation strategies [28]. The construction of a comfortable and reasonable animal house is one of the important conditions for the captive survival and health of giant pandas, and the control of the indoor environmental quality is directly related to the health of the giant pandas. Some scholars have paid attention to the need to set up regulating measures to reduce heat stress in giant pandas during high temperatures in summer [29]. However, this study shows that even though giant pandas are believed to tolerate cold environments, hemorheology is often a forgotten factor in vascular health. The risks of changes in hemorheology in giant pandas during the winter (cold) period and their clinical relevance to vascular diseases should still be considered when housing and feeding them. Future research directions may include the following aspects: exploring the impact of different ages and seasons on the metabolic activity of captive giant pandas and further studying the activity changes and differences in the overall metabolic processes of giant pandas in different age groups to understand their physiological and metabolic needs at different stages and find suitable feeding and management methods for giant pandas of different age groups. At the same time, continuing to explore the effects of factors such as seasons and climate on giant pandas, and whether these factors may lead to environmental stress, thus affecting the health and survival status of giant pandas, is warranted. In-depth study of the relationship between blood viscosity and the health condition of giant pandas would be beneficial, necessitating analysis of the negative effects of blood viscosity on giant pandas and exploring the relationship between blood viscosity and the health condition of giant pandas. Monitoring and analyzing the changes in blood viscosity of giant pandas over a long period of time can help to identify the risk of giant pandas getting sick early and take corresponding treatment and preventive measures. Studying the effects of environmental factors on the physiology and behavior of giant pandas to understand further the effects of environmental factors such as temperature, humidity, and lighting on the physiology and behavior of giant pandas is another area that could be further explored. By controlling and improving the environment of giant pandas, a more suitable captive environment can be provided.

## 5. Conclusions

This study provides a baseline for future blood sample comparisons in captive giant pandas under non-anesthetic conditions. It also reveals the impact of age and season on blood parameters, hemorheology, and biochemical parameters in captive pandas. Age-related changes may be indicative of aging and reduced metabolic activity, while seasonal variations appear to have a regulatory effect on blood viscosity. Further research is needed to investigate whether factors such as season and climate contribute to environmental stress in captive giant pandas, as it has a potentially negative impact on blood viscosity.

## Figures and Tables

**Table 1:** Basic information about giant pandas in captivity.

Name	Sex	Age	Pedigree Number	Birthday
Dadi	♂	28	394	1992.9.22
Jini	♀	27	403	1993.11.4
Gugu	♂	21	496	1999.9.25
Mengmeng	♀	14	652	2006.9.13
Fulu	♀	7	883	2013.8.8
Mengda	♂	7	894	2013.8.22
Menger	♂	7	895	2013.8.22
Diandian	♀	5	946	2015.6.20
Menglan	♂	5	954	2015.7.5
Fuxing	♂	3	1072	2017.6.25
Mengbao	♀	2	1122	2018.5.23
Mengyu	♀	2	1123	2018.5.23

**Table 2:** Characteristics of giant pandas.

Variables	Total	Sub-Adult	Adult	Old
n	12	5	4	3
Age (Year)	10.67 ± 9.12	2.33 ± 0.47	6.2 ± 0.98	22.50 ± 5.59
Sex (Male/Female)	6/6	2/3	2/2	2/1
Weight (Kg)	94.58 ± 8.59	90.63 ± 9.01	96.8 ± 7.6	96.17 ± 8.99

**Table 3:** Outdoor living environment temperature and body temperature of giant pandas (Mean ± SEM).

Variables	Spring	Summer	Autumn	Winter
Temperature (°C)	14.17 ± 4.19	25.67 ± 9.12 ^A^	12.83 ± 5.93	−0.5 ± 1.5 ^B^
Body temperature (°C)	36.6 ± 0.3	37.2 ± 0.4	36.5 ± 0.5	35.8 ± 0.5

^A,B^ Values within a row with different superscripts differ significantly at *p* < 0.01.

**Table 4:** The hematological parameters of captive giant pandas (Mean ± SEM).

		Hemoglobin (g/L)	WBC (10^9^/L)	RBC (10^12^/L)	Hematocrit (L/L)
Season	Spring	138.87 ± 4.50 ^a^	8.17 ± 0.49	7.82 ± 0.38 ^a^	0.44 ± 0.01
	Summer	125.84 ± 4.50 ^b^	8.54 ± 0.64	6.83 ± 0.28 ^b^	0.43 ± 0.01
	Autumn	120.87 ± 3.27 ^b^	7.49 ± 0.58	6.50 ± 0.24 ^b^	0.45 ± 0.01
	Winter	139.19 ± 7.05 ^a^	9.52 ± 0.50	7.76 ± 0.45 ^a^	0.46 ± 0.01
Age	Old	127.50 ± 4.92 ^b^	7.32 ± 0.38 ^b^	7.29 ± 0.34	0.39 ± 0.01 ^B^
	Adult	136.47 ± 5.18 ^a^	9.74 ± 0.59 ^a^	7.68 ± 0.44 ^a^	0.45 ± 0.01 ^A^
	Sub-adult	133.37 ± 3.73 ^ab^	8.57 ± 0.47 ^b^	7.04 ± 0.24 ^b^	0.45 ± 0.01 ^A^
*p*-value	Season	0.004	0.06	0.02	0.07
	Age	0.03	0.007	0.04	<0.001
	Interaction	0.09	0.38	0.88	0.77

^a,b^ Values within a row with different superscripts differ significantly at *p* < 0.05. ^A,B^ Values within a row with different superscripts differ significantly at *p* < 0.01.

**Table 5:** The hemorheological parameters in captive giant pandas (Mean ± SEM).

		LBV (mPa·S)	MBV (mPa·S)	HBV (mPa·S)	PV (mPa·S)	ESR (mm/H)	K Value	Fibrinogen (g/L)	EAI	EDI	ERI
Season	Spring	12.76 ± 0.30	6.71 ± 0.16	5.38 ± 0.13	1.69 ± 0.04 ^aA^	15.04 ± 0.15	59.17 ± 1.37	3.30 ± 0.08	7.63 ± 0.23 ^bAB^	0.83 ± 0.01	4.96 ± 0.18 ^bB^
	Summer	12.27 ± 0.55	6.45 ± 0.29	5.17 ± 0.23	1.82 ± 0.03 ^bcAB^	15.32 ± 0.24	56.71 ± 2.55	3.41 ± 0.13	6.73 ± 0.23 ^aA^	0.79 ± 0.01	4.24 ± 0.14 aA
	Autumn	13.18 ± 0.31	6.92 ± 0.16	5.55 ± 0.13	1.86 ± 0.04 ^cB^	15.13 ± 0.24	60.16 ± 2.08	3.61 ± 0.12	7.14 ± 0.22 ^abAB^	0.80 ± 0.02	4.51 ± 0.18 ^abAB^
	Winter	13.55 ± 0.34	7.12 ± 0.17	5.71 ± 0.14	1.74 ± 0.03 ^abAB^	14.81 ± 0.14	62.80 ± 1.59	3.51 ± 0.07	7.82 ± 0.21 ^bB^	0.82 ± 0.01	4.98 ± 0.12 ^bB^
Age	Old	12.08 ± 0.56	6.42 ± 0.24	7.51 ± 2.23	2.24 ± 0.45	18.11 ± 2.92	57.92 ± 1.52	3.42 ± 0.08	7.07 ± 0.23	0.80 ± 0.01	4.49 ± 0.17
	Adult	13.11 ± 0.42	6.88 ± 0.22	5.52 ± 0.18	1.75 ± 0.04	15.05 ± 0.22	59.78 ± 2.24	3.42 ± 0.13	7.52 ± 0.22	0.83 ± 0.02	4.87 ± 0.16
	Sub-adult	13.07 ± 0.32	6.86 ± 0.17	5.51 ± 0.14	1.76 ± 0.03	15.00 ± 0.13	60.76 ± 1.55	3.48 ± 0.082	7.44 ± 0.20	0.81 ± 0.01	4.73 ± 0.13
*p*-value	Season	0.18	0.18	0.18	0.008	0.45	0.23	0.21	0.007	0.11	0.004
	Age	0.16	0.16	0.16	0.69	0.10	0.11	0.52	0.09	0.81	0.23
	Interaction	0.93	0.93	0.92	0.89	0.63	0.93	0.96	0.59	0.65	0.55

Abbreviations: WBLSV = whole blood low-shear viscosity; WBMSV = whole blood moderate-shear viscosity; WBHSV = whole blood high-shear viscosity; PV = plasma viscosity; ESR = erythrocyte sedimentation rate; EAI = erythrocyte aggregation index; EDI = erythrocyte deformation index; ERI = erythrocyte rigidity index. ^a–c^ Values within a row with different superscripts differ significantly at *p* < 0.05. ^A,B^ Values within a row with different superscripts differ significantly at *p* < 0.01.

**Table 6:** The serum enzymes of giant pandas (Mean ± SEM).

		ALT (U/L)	AST (U/L)	Cholinesterase (U/L)	GGT(U/L)	ALP (U/L)	ADA (U/L)	Amylase (U/L)	Lipase (U/L)	CK (U/L)	CK-MB (U/L)	LD (U/L)	α-HBDH (U/L)
Season	Spring	59.48 ± 3.90 ^b^	54.49 ± 3.93	980.20 ± 73.67 ^b^	4.63 ± 0.54	160.18 ± 15.91 ^b^	25.01 ± 3.33	988.11 ± 42.46	12.30 ± 1.38 ^b^	116.78 ± 7.69	116.78 ± 7.69	978.30 ± 80.09	900.38 ± 60.22
	Summer	49.01 ± 3.43 ^ab^	53.55 ± 4.16	745.42 ± 60.24 ^a^	4.48 ± 0.49	123.47 ± 17.39 ^ab^	19.09 ± 2.33	989.94 ± 83.98	7.28 ± 1.01 ^a^	129.74 ± 8.82	129.74 ± 8.82	883.41 ± 87.28	769.33 ± 62.44
	Autumn	43.98 ± 3.59 ^a^	50.17 ± 2.57	799.82 ± 36.21 ^ab^	4.18 ± 0.64	110.26 ± 11.86 ^a^	20.08 ± 2.42	896.46 ± 68.60	7.76 ± 0.57 ^a^	103.49 ± 9.46	103.49 ± 9.46	913.77 ± 68.39	779.60 ± 56.18
	Winter	55.22 ± 5.06 ^ab^	55.07 ± 5.23	875.84 ± 105.35 ^ab^	5.65 ± 0.91	163.75 ± 16.61 ^b^	19.19 ± 2.58	1167.40 ± 122.34	10.98 ± 1.88 ^ab^	110.75 ± 8.16	110.75 ± 8.16	771.27 ± 106.53	752.15 ± 69.40
Age	Old	44.83 ± 1.49 ^aA^	67.72 ± 3.96 ^cB^	735.79 ± 52.13 ^aA^	4.83 ± 0.61	104.57 ± 7.26 ^aA^	27.22 ± 2.88 ^bB^	1072.70 ± 40.76	756.74 ± 55.51	115.91 ± 7.84	183.10 ± 13.07 ^ab^	894.36 ± 60.52 ^aA^	756.74 ± 55.51 ^aA^
	Adult	67.69 ± 5.02 ^bB^	55.90 ± 3.89 ^bB^	782.68 ± 35.37 ^aA^	3.18 ± 0.31	106.71 ± 8.26 ^aA^	25.07 ± 2.59 ^bB^	1086.73 ± 74.83	1042.02 ± 37.00	102.98 ± 7.24	149.73 ± 9.97 ^a^	1209.04 ± 52.22 ^bB^	1042.02 ± 37.00 ^bB^
	Sub-adult	51.20 ± 2.41 ^aA^	41.75 ± 1.69 ^aA^	1094.67 ± 39.53 ^bB^	5.66 ± 0.49	196.18 ± 12.05 ^bB^	14.50 ± 1.25 ^aA^	950.70 ± 72.91	708.74 ± 44.25	122.85 ± 7.85	199.50 ± 12.91 ^b^	834.56 ± 53.57 ^aA^	708.74 ± 44.25 ^aA^
*p*-value	Season	0.001	0.12	<0.001	0.65	0.01	0.15	0.16	0.01	0.13	0.43	0.38	0.13
	Age	<0.001	<0.001	<0.001	0.13	<0.001	<0.001	0.07	0.17	0.20	0.04	<0.001	<0.001
	Interaction	0.14	0.04	0.31	0.80	0.49	0.81	0.69	0.88	0.31	0.66	0.95	0.94

Abbreviations: ALT  =  alanine transaminase; AST  =  aspartate aminotransferase; GGT  =  γ-glutamyl transpeptidase; ALP  =  alkaline phosphatase; ADA = adenosine deaminase; CK  =  creatinine kinase; LD  =  lactate dehydrogenase; α-HBDH = hydroxybutyrate dehydrogenase. ^a–c^ Values within a row with different superscripts differ significantly at *p* < 0.05. ^A,B^ Values within a row with different superscripts differ significantly at *p* < 0.01.

**Table 7:** The serum proteins of giant pandas (Mean ± SEM).

		Total Protein (g/L)	Albumin (g/L)	Globulin (g/L)
Season	Spring	61.55 ± 1.13	29.08 ± 1.24	33.47 ± 1.87
	Summer	58.64 ± 1.44	29.00 ± 1.46	29.63 ± 2.26
	Autumn	64.04 ± 2.48	26.27 ± 2.07	36.66 ± 3.52
	Winter	61.44 ± 1.78	30.90 ± 1.35	30.68 ± 2.38
Age	Old	62.72 ± 1.58 ^a^	26.72 ± 1.24	36.34 ± 2.37 ^b^
	Adult	60.54 ± 3.32 ^a^	30.04 ± 1.43	32.87 ± 2.60 ^ab^
	Sub-adult	58.66 ± 1.07 ^b^	29.85 ± 1.26	28.80 ± 1.48 ^a^
*p*-value	Season	0.18	0.46	0.24
	Age	0.01	0.07	0.008
	Interaction	0.36	0.66	0.83

^a,b^ Values within a row with different superscripts differ significantly at *p* < 0.05.

**Table 8:** The serum metabolites of giant pandas (Mean ± SEM).

		Bile Acids (mmol/L)	Triglycerides (mmol/L)	Bilirubin (Total) (μmol/L)	Bile Acids (μmol/L)	Glucose (mmol/L)	Creatinine (μmol/L)	Urea (mmol/L)	Uric Acid (μmol/L)
Season	Spring	4.20 ± 0.23	1.53 ± 0.16	0.58 ± 0.05	40.51 ± 4.18	3.88 ± 0.17	93.53 ± 5.87	4.61 ± 0.40	56.78 ± 2.55
	Summer	4.67 ± 0.25	1.64 ± 0.20	0.54 ± 0.07	54.54 ± 5.64	3.63 ± 0.16	114.18 ± 7.64	4.66 ± 0.41	49.88 ± 2.78
	Autumn	4.16 ± 0.23	1.60 ± 0.24	0.84 ± 0.13	39.04 ± 4.84	3.88 ± 0.35	115.22 ± 10.55	5.69 ± 0.49	50.60 ± 3.84
	Winter	4.12 ± 0.20	1.26 ± 0.19	0.70 ± 0.09	43.09 ± 3.640	4.33 ± 0.17	96.73 ± 6.94	5.52 ± 0.56	56.17 ± 3.53
Age	Old	4.20 ± 0.20 ^ab^	1.78 ± 0.16 ^a^	0.73 ± 0.12	42.36 ± 2.96 ^ab^	3.46 ± 0.12	115.14 ± 7.56	5.32 ± 0.43	41.86 ± 2.50 ^aA^
	Adult	3.88 ± 0.18 ^a^	0.97 ± 0.06 ^b^	0.88 ± 0.20	35.50 ± 3.89 ^a^	4.00 ± 0.30	95.60 ± 6.75	4.30 ± 0.40	59.56 ± 3.48 ^bB^
	Sub-adult	4.59 ± 0.20 ^b^	1.69 ± 0.17 ^a^	0.66 ± 0.08	51.35 ± 4.78 ^b^	4.26 ± 0.14	97.39 ± 6.43	5.10 ± 0.38	57.86 ± 1.62 ^bB^
*p*-value	Season	0.23	0.59	0.33	0.09	0.14	0.20	0.25	0.05
	Age	0.01	0.03	0.67	0.04	0.10	0.19	0.38	<0.001
	Interaction	0.78	0.82	0.31	0.83	0.34	>0.99	0.16	0.92

^a,b^ Values within a row with different superscripts differ significantly at *p* < 0.05. ^A,B^ Values within a row with different superscripts differ significantly at *p* < 0.01.

## Data Availability

Not applicable.
